# Common Ancestry from Southern Italy: Two Families with Dilated Cardiomyopathy Share the Same Homozygous Loss-of-Function Variant in *NRAP*

**DOI:** 10.3390/genes16121470

**Published:** 2025-12-08

**Authors:** Maria Elena Onore, Martina Caiazza, Catia Mio, Gioacchino Scarano, Pasquale Di Letto, Sarah Iffat Rahman, Emanuele Monda, Cristiano Amarelli, Rossella Nicoletta Borrelli, Flavio Faletra, Vincenzo Nigro, Giuseppe Limongelli, Giulio Piluso

**Affiliations:** 1 Department of Precision Medicine, University of Campania “Luigi Vanvitelli”, 80138 Naples, Italy; maria.elena.onore@gmail.com (M.E.O.); pasqualediletto17@gmail.com (P.D.L.); sarah.iffatrahman@unicampania.it (S.I.R.); ronib@hotmail.it (R.N.B.); vincenzo.nigro@unicampania.it (V.N.); 2 Inherited and Rare Cardiovascular Diseases Unit, Department of Translational Medical Sciences, University of Campania “Luigi Vanvitelli”, Monaldi Hospital, 80131 Naples, Italy; martina.caiazza@yahoo.it (M.C.); gioac.scarano51@gmail.com (G.S.); emanuele.monda@unicampania.it (E.M.); giuseppe.limongelli@unicampania.it (G.L.); 3 Department of Medicine (DMED), University of Udine, 33100 Udine, Italy; catia.mio@uniud.it (C.M.); flavio.faletra@asufc.sanita.fvg.it (F.F.); 4 U.O.S.D. Genetica Medica, A.O.R.N. San Pio, 82100 Benevento, Italy; 5 Unit of Innovative Procedures in General Cardiac Surgery and Transplantation, Department of Cardiac Surgery, A.O.R.N. Ospedale dei Colli, 80131 Naples, Italy; cristiano.amarelli@ospedalideicolli.it; 6 Institute of Medical Genetics, Azienda Sanitaria Universitaria Friuli Centrale (ASUFC), 33100 Udine, Italy; 7 Telethon Institute of Genetics and Medicine (TIGEM), 80078 Pozzuoli, Italy; 8 Institute of Cardiovascular Science, University College, London WC1E 6BT, UK; 9 St. Bartholomew’s Hospital, London EC1A 7BE, UK

**Keywords:** *NRAP*, Nebulin-related anchoring protein, homozygous loss of function variants, dilated cardiomyopathy

## Abstract

**Background:** Cardiomyopathies are a heterogeneous group of heart muscle disorders with diverse genetic origins. Biallelic loss-of-function (LoF) variants in the nebulin-related anchoring protein (*NRAP*) gene have been linked to dilated cardiomyopathy (DCM) and left ventricular noncompaction cardiomyopathy, though only a few families have been described. NRAP, a member of the Nebulin family, plays a key role in cardiomyocyte development, structural integrity, and muscle function. **Methods:** We investigated two Italian siblings with DCM born to consanguineous parents from a small village in Campania. Exome sequencing, homozygosity mapping, and comparative analyses with other reported cases were performed. Genealogical research was conducted using civil registry data to reconstruct extended family pedigrees. **Results:** Both siblings were homozygous for a LoF variant in *NRAP* (NM_198060.4:c.619del; p.Val207TrpfsTer20). A third brother with tachycardia-induced cardiomyopathy, as well as their living mother, who did not have cardiac abnormalities, were found to be heterozygous. The same homozygous variant was recently identified in another Italian family with DCM coming from North-eastern Italy, whose proband also originated from a nearby village in Campania. These two families exhibited heterogeneity in clinical presentation. Homozygosity analysis revealed a >25 Mb shared region on chromosome 10 encompassing *NRAP*, supporting a common ancestral origin. While genealogical reconstruction did not allow identification of a shared ancestor, it confirmed consanguinity and enabled the recognition of potential carriers across both families. **Conclusions:** Our findings strengthen the evidence for *NRAP* as a disease-causing gene in cardiomyopathies and highlight a likely founder effect in Campania. Incorporating *NRAP* into genetic testing panels is warranted, especially in populations with high rates of consanguinity or suspected founder variants.

## 1. Introduction

Cardiomyopathies are a heterogeneous group of heart muscle diseases characterized by structurally and functionally abnormal myocardium. Classified by the European Society of Cardiology (ESC) in five different clinically phenotypes–hypertrophic (HCM), dilated (DCM), non-dilated left ventricular (NDLVC), arrhythmogenic right ventricular (ARVC), and restrictive (RCM) [1]—the cardiomyopathies are considered a common cause of premature sudden cardiac death. The genetic background is highly heterogeneous, and several genes are known to be involved in cardiomyopathies, most of them encoding for sarcomeric, desmosomal and ion channel proteins, such as *MYH7*, *MYBPC3*, *TNNT2*, *TNNI3*, *TPM1*, *TTN*, *LMNA*, *DSP*, *PKP2*, *SCN5A* [2]. Recently, mutations in a candidate gene, *NRAP*, have been reported in patients with DCM and left ventricular non-compaction (LVNC) [3,4,5,6,7,8,9,10,11,12].

The human gene *NRAP* encodes for the Nebulin-related anchoring protein (NRAP), a muscle-specific isoform and a member of the Nebulin family [13]. Nebulin is widely expressed in skeletal muscle and is only detectable at low levels in the cardiac muscle. It is the biggest protein of the family (600–900 kDa) that binds to the thin actin filament within the sarcomeres. The other members of the family (NRAP, Nebulette, LASP-1 and LASP-2) differ in their molecular weight due to different domain organization and composition [13,14].

Encoded by different genes, the members of the Nebulin family all share an actin-binding domain named the Nebulin repeat, a stretch of 35 amino acids containing a central conserved SDxxYK motif [15]. The number of Nebulin repeats differs between all the family members (Nebulin *n* = 186; Nebulette *n* = 23; LASP-1 *n* = 1; LASP-2 *n* = 3; NRAP *n* = 11). Nebulin family members also differ in the unique combination of protein motifs. The N-terminal glutamate-rich region is only present in Nebulin and Nebulette, whereas LASP-1, LASP-2 and NRAP share an N-terminal cysteine-rich LIM domain. Except for NRAP, other members contain a serine-rich region and a C-terminal SH3 domain [14].

NRAP is a 197 kDa protein that contains 11 Nebulin repeats and the N-terminal cysteine-rich LIM domain. Like in Nebulin, the Nebulin repeats are organized into a Nebulin super-repeat, a group of seven single repeats with a conserved WLKGIGW motif [16]. This motif, located at the end of the third repeat within each super-repeat, is supposed to interact with troponin/tropomyosin complexes along the actin filament. Nebulin contains 22 super-repeats, while NRAP consists of only five super-repeats [17]. Different NRAP interactions with several proteins have been described in the literature [18]. With the N-terminal cysteine-rich LIM domain, NRAP interacts with α-actinin and talin [18], while Nebulin single repeats are involved in interactions with actin, α-actinin and KLHL41 (the Kelch-like family member 41), as well as CSRP3 (cysteine and glycine-rich protein 3) and MLP (muscle LIM protein). NRAP also interacts with filamin C and vinculin using the C-terminal super-repeats [19].

NRAP exists in two different isoforms, C and S, with different expression in the heart and skeletal muscle. In the heart, only the isoform C is expressed, while both isoforms are expressed in skeletal muscle, with a lower amount of isoform C [17].

In mice, expression and localization of NRAP have been extensively investigated during skeletal muscle development, and it is localized to intercalated discs of cardiomyocytes and myotendinous junctions of skeletal muscle [20].

Pathogenic homozygous or compound heterozygous variants in Nebulin gene (*NEB*; MIM 161650) are associated with nemaline myopathy (NEM2; MIM 256030), a non-dystrophic congenital myopathy [21]. A more severe phenotype caused by biallelic variants in *NEB* is the arthrogryposis multiplex congenita-6 (AMC6; MIM 619334) [22].

Deletions involving the Nebulette gene (*NEBL*; MIM: 605491) have been reported in patients with craniofacial dysmorphism, developmental delay, and cardiac anomalies [23].

Biallelic loss-of-function (LoF) variants of *NRAP* have been associated with DCM or LVNC, strongly supporting its involvement in human cardiomyopathies [3,4,5,6,7,8,9,11,12].

Here, we report two siblings with DCM in which exome sequencing (ES) identified a homozygous 1 bp deletion involving exon 7 of *NRAP* [NM_198060.4:c.619del; p.(Val207TrpfsTer20)]. Furthermore, genetic analysis confirmed that this family and the previously studied family from Northeastern Italy [10], both carrying the same homozygous LoF variant in *NRAP*, were inbred and likely descended from common ancestors originating from a small region in Southern Italy. Additionally, we provide a comprehensive clinical characterization of DCM patients from both families.

Together, these clinical and genetic findings improve our understanding of the phenotypic consequences of pathogenic *NRAP* variants. They also provide valuable information for molecular diagnosis in unresolved DCM cases from the same geographic area. In these cases, the role of *NRAP* and a potential founder effect should be considered.

## 2. Materials and Methods

### 2.1. Clinical Evaluation

**Figure 1:**
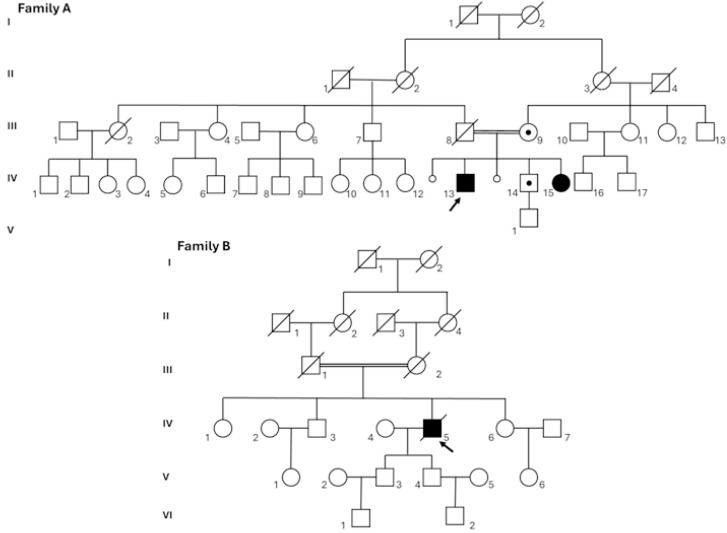
Pedigree of Families A and B. In both families, a black symbol indicates the affected individual, while an arrow indicates the proband. Genetically confirmed heterozygous carrier individuals are highlighted with a black dot.

The DCM patients from Family A and B (Figure 1) were evaluated at the Inherited and Rare Cardiovascular Diseases Unit, Department of Translational Medical Sciences, University of Campania “Luigi Vanvitelli”, Monaldi Hospital, Naples, Italy, and at the Cardiothoracic Department of the Azienda Sanitaria Universitaria Friuli Centrale (ASUFC), Udine, Italy, respectively. A full cardiological assessment and genetic evaluation of affected subjects and their relatives, when available, was performed.

### 2.2. Sample Collection

Written informed consent for blood sample collection and genetic investigation was obtained from probands and their relatives, according to the Declaration of Helsinki. The study was approved by the Institutional Review Boards of the University of Campania “Luigi Vanvitelli”. For each subject, genomic DNA was extracted using standard procedures [24] and quantified using NanoPhotometer (Implen, Munich, Germany).

### 2.3. Exome Sequencing

For Family A, the analysis was carried out only on the proband (A.IV.13) using the Agilent SureSelectXT Human All Exon V8 kit, according to the manufacturer’s instructions (Agilent Technologies, Santa Clara, CA, USA). Sequencing was performed using the Novaseq 6000 system (Illumina, San Diego, CA, USA), obtaining a mean coverage of targeted regions of 98.6% at 20x, with high sensitivity and specificity in variant detection. Sequence reads were mapped to the reference human genome assembly (December 2013, GRCh38/hg38) and analyzed using an in-house pipeline. Variant calling was performed with the Genome Analysis Toolkit (GATK) (gatk.broadinstitute.org) and variants were annotated using the Ensembl Variant Effect Predictor (VEP) [25]. For filtering of variants that had passed quality controls, we considered: (1) allele frequency < 1% in global and European populations (gnomAD database); (2) absence in our internal database (~5000 exomes); (3) de novo occurrence and Mendelian inheritance patterns; (4) filtering for genes involved to cardiac diseases (virtual panels); (5) effect on gene function and predicted pathogenicity (SIFT, PolyPhen, MutationTaster, PROVEAN, and ClinVar). Variants were classified according to ACMG guidelines [26]. For the proband of Family B, analysis conditions were previously reported [10].

### 2.4. Segregation Analysis

The causative variant in *NRAP* was annotated according to HGVS nomenclature on RefSeq NM_198060.4 [27]. For family A, segregation analysis was performed on the proband and available relatives by PCR amplification of the specific exon 7 and its flanking regions. PCR products were sequenced using the BigDye™ Terminator v1.1 Cycle Sequencing Kit (Thermo Fisher Scientific, Waltham, MA, USA) and analyzed on an Applied Biosystems 3500 automated DNA sequencer (Thermo Fisher Scientific, Waltham, MA, USA). Genomic DNA from a normal individual (wild-type control) was also sequenced as a reference.

### 2.5. Kinship Estimation

Runs of homozygosity (ROHs) were determined using AutoMap v1.2 [28]. To assess genetic relatedness between individuals, we employed two tools: VCFtools v0.1.16 [29] and KING v2.2.7 (Kinship-based Inference for Genome-wide association studies) [30]. VCF files from probands of two families (A.IV.13 and B.IV.5) were analyzed using both the --relatedness and --relatedness2 options in VCFtools, which compute relatedness based on the Ajk statistic [31] and the kinship coefficient (ϕ) [30], respectively. For KING analysis, genotype data were first converted from VCF to PLINK binary format (.bed/.bim/.fam) using PLINK v1.9.0-b.8 [32] with the --make-bed option. Relatedness inference was then performed using KING’s --related parameter, which estimates pairwise kinship coefficients and identifies identity-by-descent (IBD) segments in a unified procedure. The option --rplot was also used to generate a scatter plot. All analyses were conducted using our internal dataset comprising 1944 samples representative of the general population.

### 2.6. Genealogy Investigation

To identify possible shared ancestors of Families A and B, civil status documents, such as birth, marriage, and death certificates, were consulted online at the Archives for Genealogical Research (https://antenati.cultura.gov.it/ accessed on 13 May 2025) or directly at the local State Archive. These documents were available from 1809 to 1945.

## 3. Results

### 3.1. Case Presentation

Clinical features of probands and their relatives from Families A and B are summarized in Table 1.

#### 3.1.1. Family A

The proband (Table 1; Figure 1 A.IV.13) was a 44-year-old male referred to the Inherited and Rare Cardiovascular Diseases Unit for a cardiological and genetic evaluation. His past medical history showed no traditional cardiovascular risk factors or other comorbidities.

Approximately two years before, he had been admitted to the emergency department with worsening dyspnea and diagnosed with DCM with reduced ejection fraction at a local hospital. Coronary angiography excluded the presence of obstructive coronary artery disease, and insertion of an implantable cardioverter-defibrillator (ICD) was performed.

Family history revealed that the proband’s sister (Table 1; Figure 1 A.IV.15) was diagnosed with DCM in early childhood and underwent heart transplantation at the age of 6. No further clinical information is available.

At the time of evaluation, the patient complained of mild exertional dyspnea. His home medications included dapagliflozin (10 mg/day), sacubitril/valsartan (97/103 mg BID), furosemide (50 mg), spironolactone (25 mg/day), and bisoprolol (10 mg/day). On physical examination, he appeared in good general condition. His blood pressure was 115/50 mmHg and his heart rate was 65 bpm. Clinical signs of heart failure were absent.

Electrocardiogram (ECG) showed sinus rhythm with a heart rate of 65 bpm, normal atrioventricular conduction (PR interval 160 ms), and intraventricular conduction (QRS duration of 80 ms), and non-specific repolarization abnormalities in inferolateral leads were observed. Echocardiographic assessment revealed a dilated left ventricle with globally reduced systolic function (ejection fraction 45%). The mitral and aortic valves showed mild regurgitation. Thus, genetic testing was performed (see below).

Subsequent evaluation of the proband’s brother and mother was also performed.

The brother (Table 1; Figure 1 A.IV.14) was 42 years old and did not present DCM. However, he had a history of arterial hypertension, obesity, dyslipidemia, impaired glucose tolerance, and gastroesophageal reflux disease. Approximately two months before the cardiology assessment in our center, he had been diagnosed with atrial fibrillation and started an antiarrhythmic therapy with Flecainide and an anticoagulant. On examination, his blood pressure was 125/50 mmHg and his heart rate was 150 bpm. ECG revealed atrial fibrillation with a ventricular response of 150 bpm. Transthoracic echocardiogram showed a non-dilated left ventricle with globally reduced systolic function (ejection fraction 30%), and he was diagnosed with tachycardia-induced cardiomyopathy.

The mother (Figure 1 A.III.9) was 68 years old and had a history of hypertension. Her cardiological evaluation revealed no abnormalities.

#### 3.1.2. Family B

The proband (Table 1; Figure 1 B.IV.5) was a 72-year-old patient who had been admitted to the hospital at 66 years old due to dyspnea and retrosternal chest pain, the latter exacerbated by exercise. Patient’s comorbidities included hypertension, combined hyperlipidemia and type 2 diabetes. Electrocardiogram displayed a sinus rhythm with bigeminal ventricular extrasystoles, incomplete left bundle branch block (LBBB) and non-sustained ventricular tachycardia (NSVT). Blood tests highlighted a slight increase in cardiac-specific troponin I (cTnI) levels. Echocardiographic assessment showed (i) preserved left ventricle (LV) size and thickness with hypokinesia of the interventricular septum, ejection fraction of 50% and an impairment in diastolic function; (ii) mild annuloaortic ectasia; (iii) mild mitral valve regurgitation; (iv) preserved right ventricle (RV) size and function. Moreover, cardiac MRI with late gadolinium enhancement showed a moderate-to-severe reduction in LV systolic function and focal nonischemic areas in the interventricular septum and junction points. Two years later, a non-ST-elevation myocardial infarction occurred. The electrocardiogram showed sinus bradycardia (57 bpm), ventricular extrasystoles and LBBB. Since then, symptoms rapidly progressed with an augmentation in both dyspnea and retrosternal chest pain, bradycardia (56 bpm), frequent ventricular extrasystoles, nonspecific ventricular repolarization abnormalities, and NSVT. Patient was diagnosed with heart failure with persistent cTnI and N-terminal fragment of the type-B natriuretic peptide (NT-proBNP) release. The latest echocardiography highlighted: (i) left ventricular chamber dilation with mild septal hypertrophy, low grade aortic regurgitation, and moderate left ventricular dysfunction (ejection fraction 48%); (ii) mild annuloaortic ectasia; (iii) a mild mitral valve regurgitation; (iv) a mild to moderate aortic insufficiency; (v) preserved right ventricle size with slight reduction in systolic function. cTnI and NT-proBNP values were 130.2 ng/L and 2233 ng/L, respectively. Patient exitus occurred 6 years after the first clinical presentation.

### 3.2. Molecular Diagnosis

**Figure 2:**
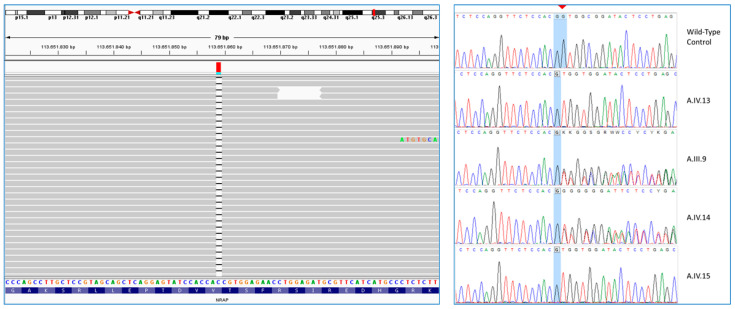
NGS data and segregation analysis of Family A—NGS data highlight the homozygous NM_198060.4:c.619del variant in *NRAP* in the proband (A.IV.13) of Family A (**left**). Segregation analysis confirms homozygosity for the affected sister (A.IV.15) of the proband, as well as heterozygosity in the carrier proband’s mother (A.III.9) and in his brother (A.IV.14) (**right**).

By exome sequencing on the proband of Family A (Figure 1 A.IV.13), we identified a homozygous 1 bp deletion in exon 7 of *NRAP* [NM_198060.4:c.619del; p.(Val207TrpfsTer20)], mapped to 10q25.3. This variant falls in the Nebulin repeat of NRAP and was predicted to be likely pathogenic according to ACMG guidelines (PVS1, PM2). The proband’s parents were consanguineous (Figure 1), and segregation analysis confirmed homozygosity in the proband because his healthy mother (A.III.9) was heterozygous for this variant. Unfortunately, it was not possible to analyze the father’s DNA as he had died before the genetic investigation. Like the proband, his affected sister (A.IV.15) resulted homozygous for the variant while his brother (A.IV.14) was heterozygous (Figure 2).

The same homozygous variant in *NRAP* was recently identified in another Italian family with DCM (Family B; Figure 1) coming from Northeastern Italy [10]. Interestingly, the proband of this family was also born in a small country in the south of Italy, in the same geographic area as Family A. Based on this observation, the relationship between these two families was genetically investigated.

### 3.3. ROHs and Kinship Estimation

**Figure 3:**
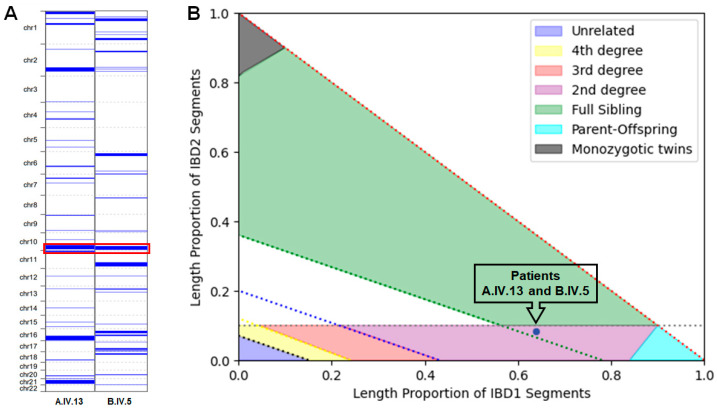
ROHs and Kinship Estimation for Families A and B—(**A**) ROHs identified by AutoMap across the genome. The red box highlights an extended ROH on chromosome 10, shared between both probands of Families A and B, encompassing the *NRAP* gene. (**B**) Scatter plot from KING illustrating pairwise relatedness based on identity-by-descent (IBD) estimates. Each colored area represents a degree of relatedness, generated with the intersections between IBD1, indicating the sharing of one allele, and IBD2, indicating sharing of both alleles at a given locus. The blue dot marks the position of the two individuals under investigation, corresponding to the probands of Families A and B.

ROHs analysis identified extensive homozygous regions in probands of two families (A.IV.13 and B.IV.5), totaling 194 Mb and 257 Mb, respectively (Figure 3A). Of note, a shared ROH of more than 25 Mb was detected on chromosome 10, encompassing *NRAP* and suggesting a potential segment of identical by descent (IBD) inheritance (Figure 3A). Excluding the homozygous LoF variant in *NRAP*, no other candidate pathogenic or likely-pathogenic variants were observed in the shared homozygous region.

The kinship analysis was performed on our internal dataset, as the underlying algorithms in both tools benefit from large cohorts for more reliable inference. For the two probands, VCFtools estimated a kinship coefficient of 0.1123 with the—relatedness option and 0.1461 with the—relatedness2 option, consistent with a second-degree relationship similar to half-siblings, grandparent–grandchild, or an avuncular (uncle–nephew) relationship [30,31]. The KING tool estimated a relatedness coefficient of 0.3247, indicating a closer genetic relationship, possibly full siblings [30] (Figure 3B). This result was also confirmed by a null value of IBS0 (identity-by-state), indicating that the two individuals share at least one allele at all analyzed loci.

To investigate the possibility of a shared ancestor between Families A and B, we performed an extensive genealogical reconstruction. Using publicly available civil registry records, we expanded each pedigree beyond the limited information provided by the probands and their relatives. This approach enabled the identification of previously unreported individuals and allowed us to extend both family trees back to approximately the early 1800s (Figure S1). Family A was traced to two adjacent, rural communities in Campania of southern Italy, each currently comprising approximately 1400–1500 inhabitants. Family B originated from a nearby small town with a population of roughly 7000. Notably, some surnames were shared between the two families, consistent with regional surname frequency and suggestive of a potential distant relationship. However, a direct genealogical link could not be confirmed, primarily due to missing data regarding the parents of the paternal great-grandfather of the proband from Family A, which limited further reconstruction of that lineage. Despite this limitation, the expanded pedigrees provided indirect evidence consistent with consanguinity and enabled the identification of additional putative heterozygous carriers in both families.

## 4. Discussion

DCM and LVNC are classified under cardiomyopathies, a diverse group of heart muscle disorders characterized by structural and functional abnormalities of the myocardium [33]. However, the most recent ESC classification does not consider LVNC to be a typical cardiomyopathy, but rather a phenotypic trait that can occur on its own or alongside other developmental abnormalities, such as ventricular hypertrophy, dilatation, and/or systolic dysfunction [1]. To date, multiple genes have been linked to DCM, including *ACTC1*, *BAG3*, *DES*, *DSG2*, *DSP*, *FLNC*, *LMNA*, *MYBPC*, *MYH6*, *MYH7*, *MYPN*, *PKP2*, *PLN*, *RBM20*, *SCN5A*, *TMPO*, *TNNT*, *TPM1*, and *TTN* [2,34].

*NRAP* is an emerging gene associated with DCM as a recessive trait, and it encodes for the Nebulin-related anchoring protein (NRAP), a muscle-specific isoform and a member of the Nebulin family [13]. Studies involving the knockdown of NRAP protein levels provide experimental evidence suggesting that reducing its levels can lead to myofibrillar disassembly in embryonic cardiomyocytes [35]. This establishes a direct role for NRAP in the early structural organization of cardiac muscle. In adult cardiac muscle, NRAP changes its primary localization and predominantly colocalizes with specialized structures such as the intercalated discs, where it acts to anchor terminal actin filaments to the sarcolemma [36]. An upregulation of NRAP was also observed in mouse models of DCM, suggesting that it may represent an adaptive response by the heart to compensate for disorganized actin thin filament architecture at intercalated disc junctions [37].

**Figure 4:**
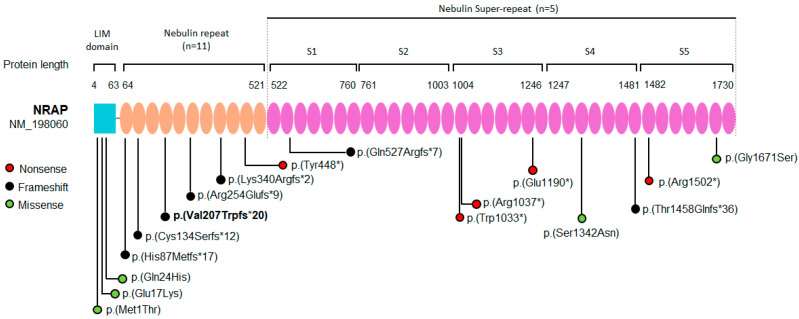
Graphical view of NRAP protein showing its functional domains and published pathogenic variants reported to date. NRAP is a 197 kDa protein composed of 1730 amino acids, and it contains an N-terminal cysteine-rich LIM domain, 11 nebulin repeats and five nebulin super-repeats. Reported variants are grouped according to their functional effect. In total, 17 *NRAP* variants have been described so far, represented by missense or truncating variants that fall within the different domains of the protein [LIM domain (*n* = 3), Nebulin repeat (*n* = 6), Nebulin super-repeat (*n* = 8)]. The variant identified in this study is highlighted in bold.

To date, only 17 distinct variants have been identified in 31 patients from unrelated families, most of whom were affected by DCM, with only two cases linked to LVNC and HCM, respectively [3,4,5,6,7,8,9,11,12]. These variants were either missense or truncating mutations (frameshift or nonsense) and were distributed across different domains of the NRAP protein, including the LIM domain (*n* = 3), Nebulin repeat (*n* = 6), and Nebulin super-repeat (*n* = 8) (Table 2 and Figure 4). Most affected individuals were homozygous for missense (*n* = 1), frameshift (*n* = 16), or nonsense (*n* = 7) variants, while the remaining cases were compound heterozygotes (Table 2). In all these families, affected individuals were born to either healthy consanguineous or unrelated parents and had mainly DCM or LVNC, with clinical manifestations occurring either in early childhood or in the third or fourth decade of life. In DCM, biallelic LoF variants of NRAP could account for about 0.25–2.46% of cases [7]. These variants lead to a reduction in NRAP protein expression, resulting in severe pathological changes in cardiomyocytes [38].

The variant identified in our index case (NM_198060.4:c.619del; p.(Val207TrpfsTer20) is located in the Nebulin repeats (Figure 4), and it was recently reported in another Italian family with DCM coming from North-eastern Italy (Family B; Figure 1) [10]. Interestingly, the proband of Family B was also born in a small country in Campania, just a few kilometers from where Family A lived. ROHs mapping and kinship estimation using different bioinformatic tools (VCFtools v0.1.16 and KING v2.2.7) on probands’ exome data from both families revealed a shared homozygous region of more than 25 Mb on chromosome 10 that included *NRAP* and strongly suggested a familial relationship (Figure 3A).

Genetic relatedness analysis indicated a strong level of shared ancestry, with approximately 25% of the genome identical-by-descent. This pattern aligns with a second-degree relationship, such as half-siblings or an avuncular connection (uncle–nephew). Interestingly, KING’s overestimation of kinship highlights a potential history of inbreeding, which could explain the increased allele sharing beyond what is expected for a typical second-degree relationship. Considering both the proportion of shared alleles and the genomic distribution of identical regions, the most plausible interpretation is that these individuals share a common ancestry influenced by endogamy, making them closely related but not immediate first-degree relatives (Figure 3B).

Although an extensive genealogical reconstruction was conducted for families A and B (Figure S1), no direct genealogical link could be established between the two families. This represents a current insurmountable limitation. Nonetheless, the expanded pedigrees provided further indirect evidence supporting consanguinity, and they may help in the identification of additional potential heterozygous carriers within both families and, possibly, in this specific geographic area of Southern Italy. This area has been rural for a long time and only experienced emigration after 1900. As illustrated by these two families (Figure S1), marriages between consanguineous individuals were not uncommon in the past. Consanguinity is frequently reported among *NRAP*-associated cardiomyopathy cases, and a founder effect has been described for the c.400_407del (p.Cys134Serfs*12) variant, which has been identified in multiple unrelated families, particularly within the Saudi Arabian population [3,4,8,12]. Recognition of such founder mutations has important implications for population-specific genetic screening and counseling, as it can enhance diagnostic efficiency and refine risk assessment within affected communities. This further underscores the importance of including *NRAP* in routine genetic testing panels for severe cardiomyopathies, often observed in infancy or early childhood, particularly in cases with suspected recessive inheritance or familial consanguinity.

In accordance with literature data [4,5,6,7,8,9,10], clinical presentations within Families A and B highlight the significant phenotypic variability that can occur even within a single family, with likely reduced penetrance only reported in two families [3,5]. In Family A, the spectrum ranges from severe early-onset DCM requiring transplantation (A.IV.15) to later-onset non-ischemic DCM (A.IV.13). Then, manifestations of *NRAP*-induced cardiac disease can differ considerably in type, severity, and age of onset.

DCM is a consistently predominant shared phenotype in patients from families A and B, as well as in the majority of previously reported *NRAP* patients [3,4,5,6,7,8,10,12]. Progression to severe heart failure is a common and often fatal outcome across severe cases, frequently necessitating advanced interventions such as cardiac transplantation or LVAD support, or leading to early mortality. Cardiac arrhythmias are also frequently observed. A significant proportion of cases linked to *NRAP* causative variants manifests in infancy or early childhood [3,6,7,8,12]. Many cases, especially those with early onset, exhibit rapid deteriorations of cardiac function, often leading to early death, while other *NRAP*-related instances (e.g., certain compound heterozygotes) [7] and the probands in Families A and B demonstrate a more stabilized course with treatment, highlighting the variability in disease progression.

*NRAP*-associated cardiomyopathies are predominantly attributable to homozygous LoF variants, with the majority of reported cases arising in the context of consanguinity. Individuals harboring homozygous LoF variants typically present with an earlier disease onset (mean age ~19 years), whereas compound heterozygotes carrying one LoF allele in combination with a missense variant manifest disease later in life (mean age ~39.5 years) (Table 2). In such cases, the presence of a missense allele is likely to preserve partial protein function, thereby mitigating disease severity and delaying onset relative to homozygous LoF genotypes. To date, the only exception is the *NRAP* p.(Gly1671Ser) homozygous missense variant, which was identified in an HCM patient with early-onset disease [8]. However, the functional impact of this missense change on protein function has not yet been investigated.

Taken together, these findings reinforce the critical role of *NRAP* in cardiomyopathy pathogenesis and highlight the importance of its inclusion in genetic testing strategies, particularly in populations with high rates of consanguinity or suspected founder variants.

## 5. Conclusions

This study offers compelling genetic evidence that substantiates a shared ancestral origin for the identified *NRAP* homozygous LoF variant in two consanguineous Italian families affected by dilated cardiomyopathy. The presence of an extended shared homozygous region on chromosome 10, encompassing *NRAP*, suggests a possible local founder effect within this geographic area. The results demonstrate how consanguinity may reveal recessive variants contributing to disease in specific family or community contexts. In light of emerging evidence that biallelic *NRAP* LoF variants are associated with recessive dilated cardiomyopathy, there is a compelling rationale for the inclusion of *NRAP* in comprehensive cardiomyopathy gene panels, particularly for patients with suspected autosomal recessive disease or a history of consanguinity.

## Figures and Tables

**Table 1:** Clinical features of Families A and B.

Feature	Family A			Family B
	Proband (A.IV.13)	Sister with DCM (A.IV.15)	Brother Without DCM (A.IV.14)	Proband (B.IV.5)
Age at onset	42 years	Early childhood; transplantation at age 6.	42 years (AF diagnosis 2 months before cardiology assessment).	66 years (first admission with dyspnea and chest pain).
Primary cardiac diagnosis	Dilated cardiomyopathy with reduced EF.	Severe DCM requiring early heart transplantation.	Tachycardia-induced cardiomyopathy in the setting of atrial fibrillation.	Progressive LV dysfunction with nonischemic injury pattern and HF.
Comorbidities	None; no traditional CV risk factors.	Not reported.	Arterial hypertension, obesity, dyslipidemia, impaired glucose tolerance, GERD.	Hypertension, combined hyperlipidemia, type 2 diabetes.
Rhythm/conduction findings	Sinus rhythm; nonspecific inferolateral repolarization changes.	Not reported.	Atrial fibrillation with ventricular response 150 bpm on ECG.	Sinus rhythm with bigeminal ventricular ectopy, NSVT; later sinus bradycardia, LBBB, frequent ventricular ectopy.
Baseline LV size and systolic function	Dilated LV with globally reduced systolic function; EF 45%.	Severe dysfunction necessitating transplant (exact EF not available).	Non-dilated LV with globally reduced systolic function; EF 30%.	Initially preserved LV size and thickness with septal hypokinesia, EF 50%; later LV dilation with moderate dysfunction, EF 48%.
Diastolic function	Not specifically reported.	Not reported.	Not reported.	Diastolic dysfunction on initial echo.
Valvular abnormalities	Mild mitral and aortic regurgitation.	Not reported.	Not reported.	Mild annuloaortic ectasia; mild mitral regurgitation; later mild–moderate aortic insufficiency and low-grade aortic regurgitation.
Ventricle size and function	Not specifically abnormal.	Not reported.	Not reported as abnormal; focus on LV dysfunction.	Preserved RV size initially; later slight reduction in RV systolic function.
Advanced imaging (CMR)	Not reported.	Not reported.	Not reported.	CMR: moderate–severe LV systolic impairment with focal nonischemic LGE in septum and junctions.
Biomarkers	Not reported.	Not reported.	Not reported.	Persistent elevation of cTnI and NT-proBNP.
Clinical course/severity	Mid-life DCM with moderate LV dysfunction, clinically stable on optimal therapy and ICD.	Malignant early childhood course with transplant at 6 years.	AF-related tachycardia cardiomyopathy with significant LV dysfunction but structurally non-dilated LV.	Adult-onset, progressively worsening HF with recurrent symptoms and arrhythmias, leading to death over 6 years.
Outcome	Alive with DCM, ICD in place, good functional status on therapy.	Survived via orthotopic heart transplantation in early childhood.	Alive; managed for AF and tachycardia-induced LV dysfunction (no DCM diagnosis).	Death 6 years after initial clinical presentation.

Notes: EF = Ejection Fraction; LV = Left Ventricle; HF = Heart Failure; CV = Cardiovascular; GERD = Gastro-esophageal reflux disease; NSVT = Non-sustained ventricular tachycardia; ECG = Electrocardiogram; LBBB = Left bundle branch block; ICD = Implantable cardioverter-defibrillator.

**Table 2:** Bi-allelic NRAP mutations (NM_198060.4).

Reference		Age at Onset, Sex	Parents	Phenotype	HGVS Nomenclature	Exon	Domain	Variant Type	Zigosity	Died (Age)	ACMG Classification
[4]	1 proband	not reported	consanguineous	DCM	c.400_407del, p.(Cys134SerfsTer12)	5	NR	Frameshift	Hom		P (PVS1, PP5, PM2)
[5]	2 related probands	26 y, M	unrelated	DCM	c.4504C > T, p.(Arg1502Ter)	38	NSR	Nonsense	Hom		LP(PP3, PM2, PP5)
		36 y, M	unrelated	asymptomatic	c.4504C > T, p.(Arg1502Ter)	38	NSR	Nonsense	Hom		LP(PP3, PM2, PP5)
[6]	1 proband	3 y 5mo, F	unrelated	DCM	c.1344T > A, p.(Tyr448Ter)	14	NR	Nonsense	Hom	3 y 8mo	P (PVS1, PM2, PP5)
[3]	1 proband and Father	13 mo, F	consanguineous	DCM	c.400_407del, p.(Cys134SerfsTer12)	5	NR	Frameshift	Hom		P (PVS1, PP5, PM2)
		33 y, M	not reported	asymptomatic	c.400_407del, p.(Cys134SerfsTer12)	5	NR	Frameshift	Hom		P (PVS1, PP5, PM2)
[12]	1 Proband	10 y, M	consanguineous	DCM	c.400_407del, p.(Cys134SerfsTer12)	5	NR	Frameshift	Hom	13 y	P (PVS1, PP5, PM2)
	1 Proband	9 mo, F	consanguineous	DCM	c.400_407del, p.(Cys134SerfsTer12)	5	NR	Frameshift	Hom	9 mo	P (PVS1, PP5, PM2)
	1 Proband	2 y, M	consanguineous	DCM	c.760del, p.(Arg254GlufsTer9)	8	NR	Frameshift	Hom	7 y	P (PVS1, PM2,PP5)
	1 Proband	22 mo, F	consanguineous	DCM	c.760del, p.(Arg254GlufsTer9)	8	NR	Frameshift	Hom	4 y	P (PVS1, PM2,PP5)
	1 Proband	12 y, F	consanguineous	DCM	c.1019del, p.(Lys340ArgfsTer2)	11	NR	Frameshift	Hom	13 y	P (PVS1, PM2,PP5)
	1 Proband	2 y, M	consanguineous	DCM	c.1579del, p.(Gln527ArgfsTer7)	16	NSR	Frameshift	Hom		P (PVS1, PM2,PP5)
	1 Proband	4 y, F	consanguineous	DCM	c.3568G > T, p.(Glu1190Ter)	31	NSR	Nonsense	Hom		P (PVS1, PM2,PP5)
[7]	11 unrelated probands	19 y, F	not reported	DCM	c.3099G > A, p.(Trp1033Ter)	28	NSR	Nonsense	Hom		P (PVS1, PM2, PP5)
		22 y, M	not reported	DCM	c.4371del, p.(Thr1458GlnfsTer36)	37	NSR	Frameshift	Hom		LP (PVS1, PM2, PP5)
		53 y, F	not reported	DCM	c.3109C > T, p.(Arg1037Ter)	28	NSR	Nonsense	Hom		P (PVS1, PM2, PP5)
		2 y, M	not reported	DCM	c.1344T > A, p.(Tyr448Ter)	14	NR	Nonsense	Hom	2 y	P (PVS1, PM2, PP5)
		36 y, F	not reported	DCM	c.4371del, p.(Thr1458GlnfsTer36)	37	NSR	Frameshift	Het Comp	38 y	LP (PVS1, PM2, PP5)
					c.72G > C, p.(Gln24His)	1	LIM	Missense			VUS (PP3, PM2, PP5, BP1)
		28 y, M	not reported	DCM	c.4371del, p.(Thr1458GlnfsTer36)	37	NSR	Frameshift	Het Comp		LP (PVS1, PM2, PP5)
					c.72G > C, p.(Gln24His)	1	LIM	Missense			VUS (PP3, PM2, PP5, BP1)
		56 y, M	not reported	DCM	c.1344T > A, p.(Tyr448Ter)	14	NR	Nonsense	Het Comp		P (PVS1, PM2, PP5)
					c.49G > A, p.(Glu17Lys)	1	LIM	Missense			VUS (PP3, PM2, BP1)
		4 y, F	not reported	DCM	c.4371del, p.(Thr1458GlnfsTer36)	37	NSR	Frameshift	Het Comp		LP (PVS1, PM2, PP5)
					c.4504C > T, p.(Arg1502Ter)	38	NSR	Nonsense			LP(PP3, PM2, PP5)
		46 y, F	not reported	DCM	c.72G > C, p.(Gln24His)	1	LIM	Missense	Het Comp		VUS (PP3, PM2, PP5, BP1)
					c.4504C > T, p.(Arg1502Ter)	38	NSR	Nonsense			LP(PP3, PM2, PP5)
		59 y, M	not reported	DCM	c.72G > C, p.(Gln24His)	1	LIM	Missense	Het Comp		VUS (PP3, PM2, PP5, BP1)
					c.4504C > T, p.(Arg1502Ter)	38	NRS	Nonsense			LP(PP3, PM2, PP5)
		48 y, M	not reported	DCM	c.4025G > A, p.(Ser1342Asn)	35	NRS	Missense	Het Comp		VUS (PM2, BP1)
					c.2T > C, p.(Met1Thr)	1	LIM	Start loss			P (PVS1, PM2, PP5)
[8]	3 unrelated probands	3 y, M	not reported	HCM	c.5011G > A, p.(Gly1671Ser)	41	NRS	Missense	Hom		VUS (PM2, PP3, BP1)
		4 y, F	consanguineous	DCM	c.400_407del, p.(Cys134SerfsTer12)	5	NR	Frameshift	Hom	5.5 y	P (PVS1, PP5, PM2)
		19 mo, M	consanguineous	DCM	c.400_407del, p.(Cys134SerfsTer12)	5	NR	Frameshift	Hom		P (PVS1, PP5, PM2)
[9]	1 proband	5 y, M	unrelated	LVNC	c.259delC, p.(His87MetfsTer17)	4	NR	Frameshift	Hom		P (PVS1, PM2, PP5)
[10]	1 proband	66 y, M	unrelated	DCM	c.619del, p.(Val207TrpfsTer20)	7	NR	Frameshift	Hom	72 y	LP (PVS1, PM2)
Family A This report	2 affected sibling	42 y, M	consanguineous	DCM	c.619del, p.(Val207TrpfsTer20)	7	NR	Frameshift	Hom		LP (PVS1, PM2)
		3 y, F		DCM	c.619del, p.(Val207TrpfsTer20)	7	NR	Frameshift	Hom		LP (PVS1, PM2)

Notes: M = male; F = female; DCM = dilated cardiomyopathy; HCM = hypertrophic cardiomyopathy; LVNC = left ventricular non-compaction cardiomyopathy; NR = Nebulin Repeat; NSR = Nebulin super-repeat; LIM = LIM domain.

## Data Availability

Additional data are available from the corresponding author on reasonable request.

## Supplementary Materials

The following supporting information can be downloaded at: https://www.mdpi.com/article/10.3390/genes16121470/s1, Figure S1: Expanded Pedigree of Families A and B.

## Abbreviations

The following abbreviations are used in this manuscript:

LoF	Loss-of-function
NRAP	Nebulin-related anchoring protein
DCM	Dilated cardiomyopathy
ESC	European Society of Cardiology
HCM	Hypertrophic cardiomyopathy
NDLVC	Non-dilated left ventricular cardiomyopathy
ARVC	Arrhythmogenic right ventricular cardiomyopathy
RCM	Restrictive cardiomyopathy
LVNC	Left ventricular non-compaction cardiomyopathy
LASP-1	LIM and SH3 Protein 1
LASP-2	LIM and SH3 Protein 2
KLHL41	Kelch-like family member 41
CSRP3	Cysteine and glycine-rich protein 3
MLP	Muscle LIM protein
GATK	Genome Analysis Toolkit
VEP	Ensembl Variant Effect Predictor
ACMG	American College of Medical genetics
HGVS	Human genome variation society
ROH	Runs of homozygosity
ECG	Electrocardiogram
LBBB	Left bundle branch block
NSVT	Non-sustained ventricular tachycardia
NT-proBNP	Type-B natriuretic peptide
IBS	Identity-by-state
LVAD	Left Ventricular Assist Device
