# Supplementary material for: Common Ancestry from Southern Italy: Two Families with Dilated Cardiomyopathy Share the Same Homozygous Loss-of-Function Variant in NRAP
Source: Genes (Basel). 2025 Dec 8;16(12):1470. doi: 10.3390/genes16121470 (PMC12732394; doi:10.3390/genes16121470)
Supplement: Supplementary file 1 [file genes-16-01470-s001.zip › genes-4021745-supplementary.pdf]

**Family A**

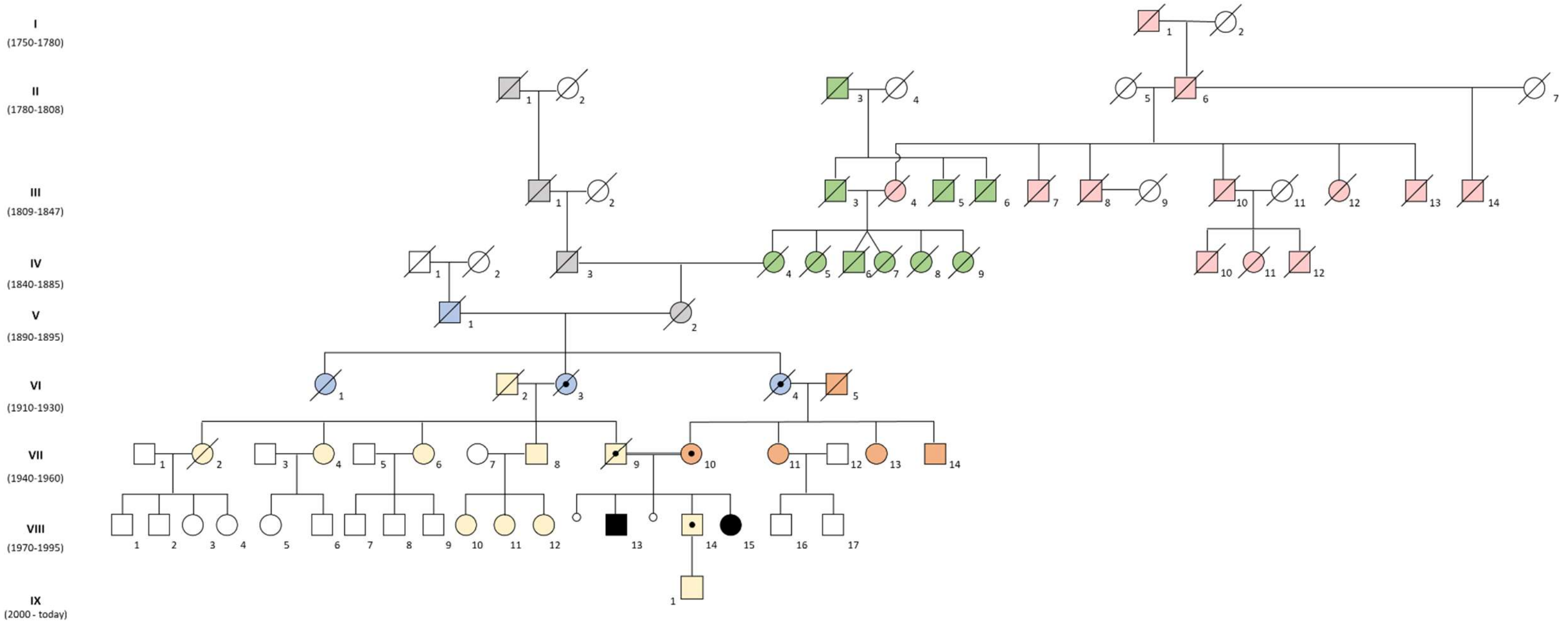


**Family B**

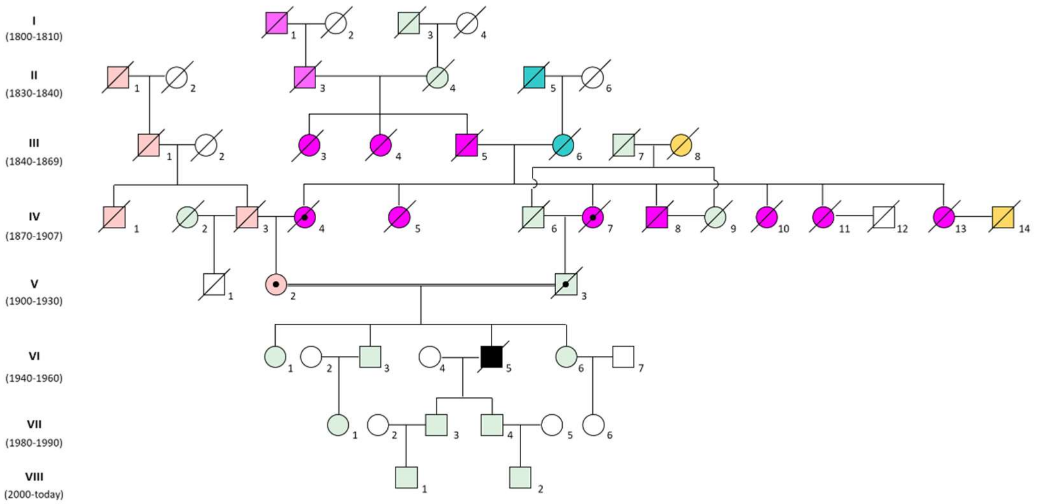


**Figure S1**- Expanded Pedigree of Families A and B

Using civil registry records, we reconstructed the pedigrees of families A and B, extending both family trees back to the early 1800s. Individuals with the same surname are highlighted in the same color in both families. For each generation, the year of birth range is also reported. Presumptive obligate carriers are identified with a black dot. In Family A, subject VIII.13 is the proband reported in Figure 1 as IV.13. In Family B, subject VI.5 corresponds to proband IV.5 in Figure 1.
